# Pharmaceutical Research - Academic Collaborations: An Insider’s Perspective

**DOI:** 10.1021/acsmedchemlett.5c00083

**Published:** 2025-04-11

**Authors:** Stevan W Djuric

**Affiliations:** Department of Pharmacology, School of Medicine, University of Virginia, Charlottesville, VA 22903, United States

**Keywords:** Academic-Industrial Collaborations, Student Training, Organic Synthesis, Enabling Medicinal Chemistry Technology, Cycle Time

## Abstract

A constant pressure on the pharmaceutical industry is the need to develop new therapeutics in a more cost-effective and timely manner. In this letter, I describe the potential that the development and implementation of enabling chemistry technology has for reducing cycle time and cost of goods. Several of these innovations were produced through industry and academic collaborations in which complementary areas of expertise were brought to the table.

## Graphical abstract

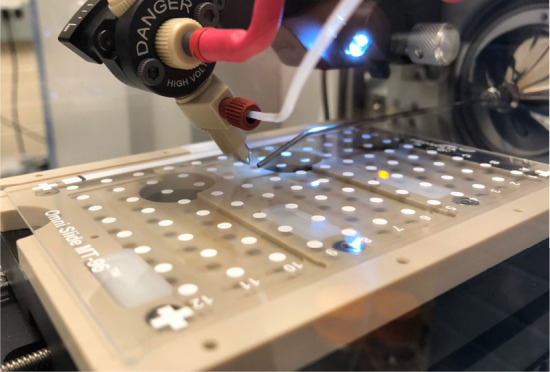

Part of my team’s charter when I was responsible for the Discovery Chemistry and Technology organization at a major pharmaceutical company was to identify, develop and implement new enabling technologies that would improve our overall success.

In considering how medicinal chemistry and organic synthesis could significantly impact our success rate we noted that Paul et al.^1^ at Lilly had proposed an algorithm (Figure 1) for improving drug discovery success by focusing on several key aspects of the process.

**Figure 1:**
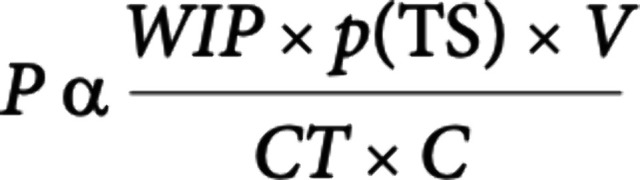
Lilly algorithm for R and D Productivity.

According to this paper, R&D productivity (P) can be viewed as a function of the elements comprising the numerator — the amount of scientific and clinical research being conducted simultaneously, designated here as the work in process (WIP), the probability of technical success (p­(TS)) and the value (V) — divided by the elements in the denominator, the cycle time (CT) and cost (C).

We felt that medicinal chemistry and new enabling chemistry technology could help significantly with two components of the algorithm a) reduction of cycle time and b) reduction in cost of goods all leading to “more and better shots on goals” with a subsequent improvement in overall probability of success.

To achieve this goal, we focused, in significant part, on opportunities to identify and implement new and improved synthetic methods that would allow us to make novel target molecules more efficiently and to introduce new enabling technologies including first in class instrumentation that would reduce cycle time and provide a competitive edge.

To this end, our internal enabling chemistry technology group developed several notable innovations for improving cycle time. These included our fully integrated Synthesis-Purify-Bioassay system (BioSIP), ChemBead technology and the use of DESI MS for rapid analysis of HTE experiments.^2^ However, due to resource and expertise constraints we were not able to conduct all technology development work in house.

To strengthen our research capabilities, we established strategic collaborations with academicians whose interests closely aligned with ours. These partnerships were built on a 50/50 research model, with jointly defined goals and milestone-based funding. While postdoctoral student sponsorships formed the cornerstone of these efforts, we also pursued additional collaborative avenues, as detailed later in this letter.

We further implemented a summer internship program, sponsoring students from collaborator laboratories to work with us over the summer. This experience provided students with valuable insights into the pharmaceutical industry, offering a practical perspective on career paths in “Big Pharma.” For projects focused on technology development, interns gained hands-on exposure to cutting-edge technologies and their implementation.

It is important to emphasize that our collaborative efforts were driven by strategic goals and tailored to our specific needs — a “fit-for-purpose” approach. What worked effectively for us might not be suitable for other organizations, reinforcing the idea that one size does not fit all.

## Collaboration Framework

The primary objective of these collaborations was to access specialized technical expertise not available in-house, which would be inefficient to develop internally. Speed and innovation were crucial, whether it involved the creation of novel synthetic methodologies to enhance high-throughput chemistry workflows or the integration of advanced instrumentation to improve efficiency and reduce labor costs.

Partner selection was meticulous. Collaborators were typically long-standing academic contacts with proven records of producing high-quality students. Trust and reliability were critical factors. Each project was overseen by a newly hired PhD medicinal chemist, typically with recent postdoctoral experience, ensuring effective coordination. Weekly teleconferences with the student and academic advisor provided consistent oversight. When possible, we conducted site visits to collaborator laboratories, and summer interns spent an entire summer in our facilities, gaining immersive industrial experience.

A secondary goal of these collaborations was to enhance our group’s visibility through increased publication output, thereby strengthening our recruiting efforts. When mutual interest and compatibility were evident, we made substantial efforts to hire these students upon completing their PhDs or postdoctoral training.

## Challenges and Adaptations

While most collaborations were successful, we did encounter setbacks. In one instance, a collaborator failed to deliver a promised set of library compounds despite multiple assurances and continued funding. This experience led us to transition from unrestricted funding models to milestone-based agreements. Under these revised contracts, collaborators received an initial payment followed by subsequent payments contingent upon the achievement of specific deliverables, such as the production of novel compounds using innovative synthetic methodologies of interest to us.

This adaptive approach not only safeguarded our investments but also ensured accountability and mutual commitment, fostering more productive and reliable collaborations.

In this brief letter, I will highlight three separate fit-for-purpose initiatives that we undertook.

### 1 Compound Collection Enhancement

Several years ago the pharmaceutical industry, as a whole, spent considerable effort and money on improving their high throughput screening collections. In our case, we chose alongside adding focused collections to our repository (e.g kinase inhibitor, GPCR antagonist, ion channel regulator), to incorporate a diverse set of drug-like molecules into the collection. As a component of this strategy, we chose to utilize Multicomponent Coupling Reactions (MCRs) incorporating a post coupling step to generate small (∼100 member) collections of compound displaying a variety of novel pharmacophores.

To achieve these goals, we entered into collaborations with both Jeff Aube’s and Paul Hanson’s groups (both then at the University of Kansas) where a student of theirs would spend an entire summer in our laboratories undertaking these activities. We have published extensively on this topic but one illustrative example with student Tim Ribelin is given here (Figure 2). In this example, we reported a concise protocol for the diastereoselective synthesis of novel bridged bicyclic lactams from commercially available components by the sequence of Ugi, ring-closing metathesis (RCM), and Heck reactions. X-ray diffraction studies revealed that the bicyclic products contain varying degrees of pyramidalization of the bridgehead nitrogen atom.^3^

**Figure 2:**
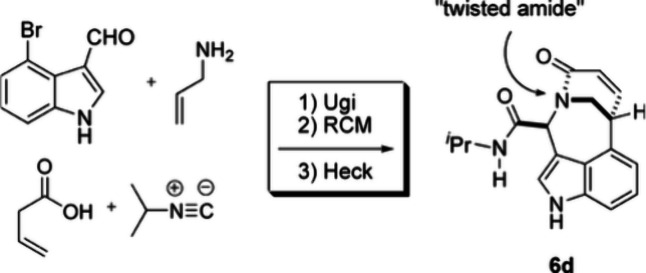
MCR chemistry Ugi-RCM-Heck protocol.

This strategy allowed for the inclusion of many completely novel structures into our screening collection. Additionally, in a similar context, we were interested in enhancing our building block/monomer collection for either high throughput chemistry or fragment-based screening activities. To this end, we forged a collaboration with Ian Baxendale at the University of Durham, in the United Kingdom where we supported a student, Michele Ruggeri, to work on the synthesis of novel azetidines, using novel flow-based Wang reaction methodology, as parallel synthesis building blocks. The appended graphic in Figure 3 highlights the transformation and concept. This example was one of many where we chose to increase our collection with novel building blocks that contained pharmacologically relevant motifs, in this case azetidines.

**Figure 3:**
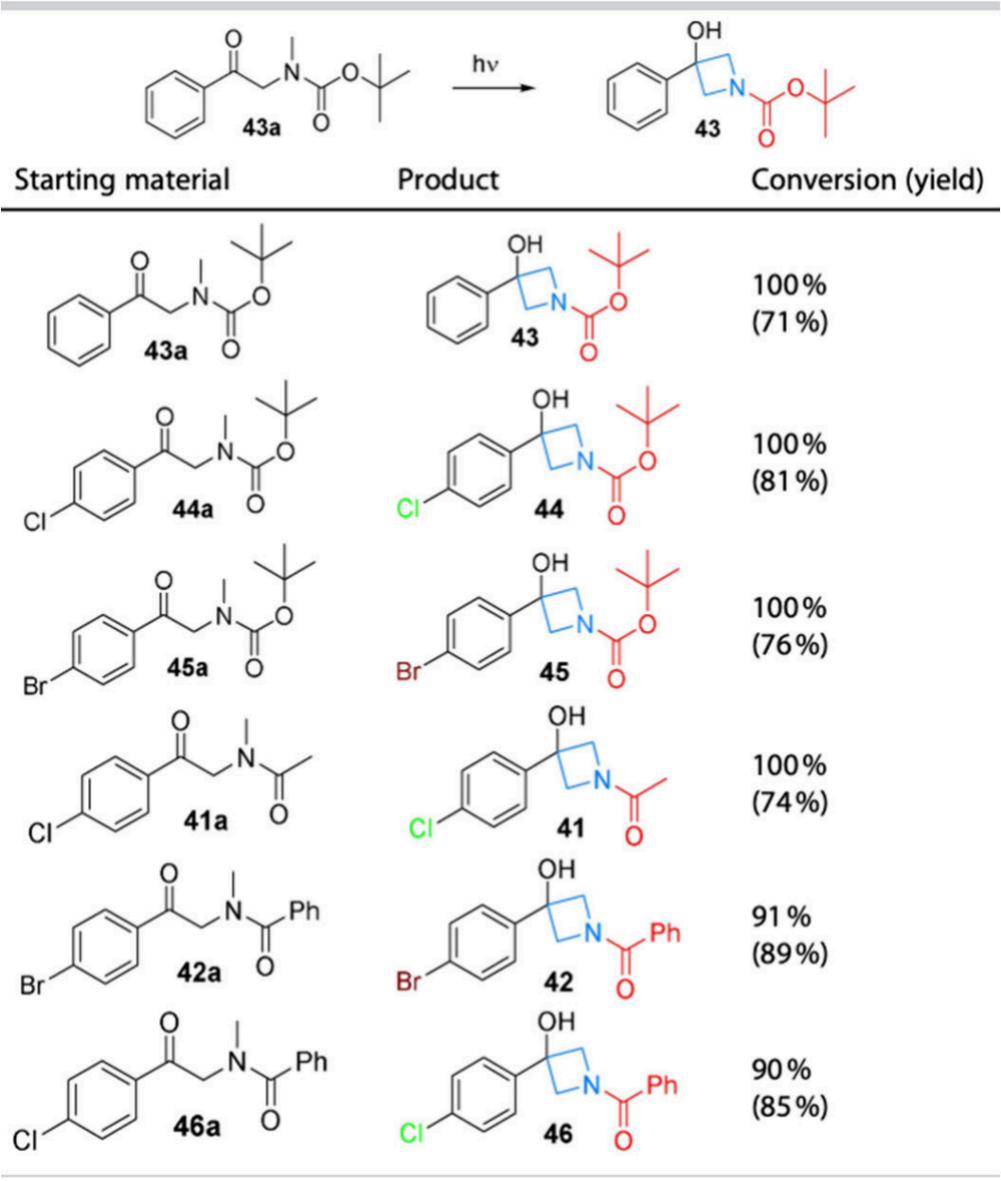
Novel building block production: representative yields from the flow enabled Yang reaction.

Azetidines represent an important heterocyclic class comprising valuable chemical and biological features synonymous with the structurally related β-lactams. Their general popularity is due to their small and highly geometrically configured structure that is very useful for exploring chemical space, making the azetidine ring a highly prized template. In these studies^4^ the Yang reaction was studied in flow for the first time; starting from a sulfonamide derivative whereby the reactivity in batch was known. The reaction was optimized in a flow reactor showing a remarkable reduction in the reaction time from several hours (batch) to minutes. Several *N*-carbonyl derivatives were subsequently assembled showing a good reactivity toward the cyclization, moreover the BOC protected azetidine particularly represents a valuable substrate giving the chance for deprotection and the use of the free nitrogen for further reactions. The reaction was also scaled up allowing the production of >20 g of product in under 12 h demonstrating the efficiency of the Yang reaction in flow as a valuable synthetic tool.^4^

This collaboration not only provided novel monomers for our collection but also garnered us significant knowledge and expertise in flow chemistry from an acknowledged leader in the area.

### 2 Enabling Chemistry Technology Development: Flow Photochemistry and Flow Electrochemistry

Aside from the desire to access novel building blocks for the reasons mentioned above, we also wanted to add to our internal work using flow photochemical (and electrochemical) reactors as part of our enabling chemistry technology initiative. This had led to the development of an internally invented and developed automated flow chemical reactor LOPHTOR.^5^ Our collaboration with the Baxendale group, as highlighted above, was part of the initiative that allowed us to gain significant expertise in flow-based reaction technology.

We had published an early flow based sequential Ugi/[2 + 2] Ene-Enone photocycloadditions this time with Alan Whitehead as the summer intern.^6^ One of the main advantages offered by flow-photochemistry is that only small unit volumes are propagated through the small dimensional flow reactor channel(s) meaning a high and uniform incident photon flux can be achieved while avoiding the inherent thermal heating effects which accompany classical batch based photochemical set-ups. Furthermore, the continuous operation characteristic of a flow process allows for a more consistent and simpler scaleup of the reaction by simply extending processing times.

On the flow electrochemistry front, we worked with Syrris to develop the first commercially available flow electrochemical reactor. Our goal, again fit for purpose, was to develop a technology whereby we could execute late-stage oxidations and fluorinations of molecules. Development of the reactor technology was done in Greg Roth’s group at the Sanford -Burnham Institute in Florida. As part of this collaboration, we sent one of our Chemistry Technology scientists, Hannes Koolman down to Florida to work in Greg’s group for a month or so in order to learn how to effectively utilize the reactor and then bring that knowledge back in-house. Some of this collaborative work was published.^7^

We also arranged for another flow electrochemistry enabled collaborative project with Shannon Stahl at the University of Wisconsin. The goal of this effort was to develop methods to rapidly access alpha-cyanoamines as peptidase inhibitors with particular reference to the DPPIV inhibitor area.

This collaboration involved staff and equipment sharing activities and was notably successful in producing a significant collection of building blocks and novel fragments for us. Once again, this work was published^8^ and summarized in Figure 4. This collaboration involved staff and equipment sharing activities and was notably successful in producing a significant collection of building blocks and novel fragments for us.

**Figure 4:**
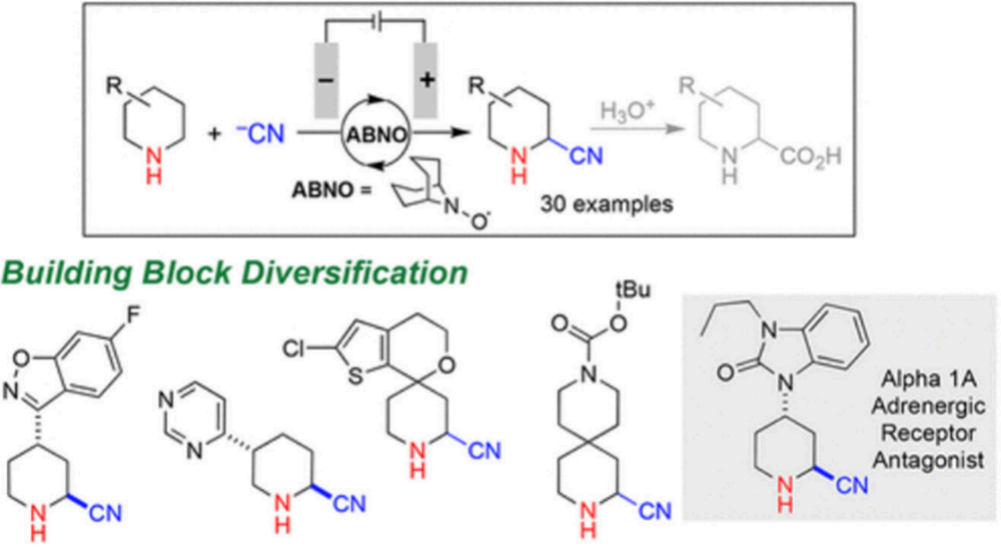
Synthesis of “Privileged” Building Blocks: Electrochemical synthesis of alpha-cyanoamines.

### 3 Technology Development for Reaction Rate Enhancement

We also developed a number of collaborations to examine the potential for developing reactors that would help accelerate reaction rates. Following from the Gibbs Free Energy equation, we implemented a program using high temperature flow reactors which ultimately, after a lot of learning turned to to be quite successful. Much of this work was done by summer intern Manwika Charaschanya from Jeff Aube’s group and postdoc Jennifer Tsoung fresh from a degree in Mark Lauten’s group in Toronto. We published extensively on this, but a pertinent example follows:

Fused pyrimidinone and quinolone derivatives that are of potential interest to pharmaceutical research were synthesized within minutes in up to 96% yield in an automated Phoenix high-temperature (provided by ThalesNano) and high-pressure continuous flow reactor. Heterocyclic scaffolds that are either hard to synthesize or require multisteps were readily accessible using a common set of reaction conditions as depicted in Figure 5. The use of low-boiling solvents along with the high conversions of these reactions allowed for facile workup and isolation. The methods reported were highly amenable for fast and efficient heterocycle synthesis as well as compound scale-ups.^9^

**Figure 5:**
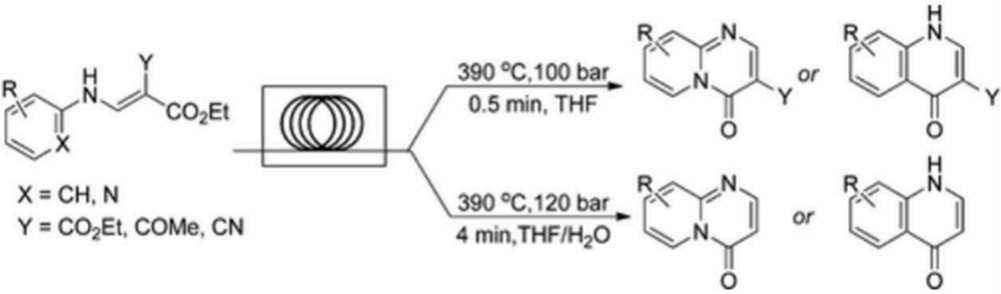
High temperature enabled flow synthesis of key heterocycles.

This effort not only allowed use to identify a useful technology but also gained useful experience in working with supercritical fluids.

One important point to note here is that, to paraphrase a George Whitesides quote, if a technology has value people will buy it. In our case, the technologies that the group had developed had to be widely embraced by the broader chemistry community at the company. If not, then the particular initiative could be deemed unsuccessful.

In a more exploratory vein, we had become intrigued by the potential for reaction acceleration using micro droplets and initiated a collaboration with Graham Cooks group at Purdue who were working along similar lines (as was the Zare group at Stanford).

In this work,^10^ electrospray (ES) and paper spray (PS) mass spectrometry were used to create confined liquid volumes in which accelerated air and water sensitive, heterogeneous, copper catalyzed C–O and C–N coupling reactions occur. Significant reaction acceleration was observed compared to the bulk reaction which required elevated temperatures and time for completion. Macroscopic amounts of product (mg scale) were prepared using offline ES within minutes. The trends in reactivity observed for several reagents matched those of the bulk reactions making droplet accelerated reactions good mimics of the bulk chemistry (Figure 6)

**Figure 6:**
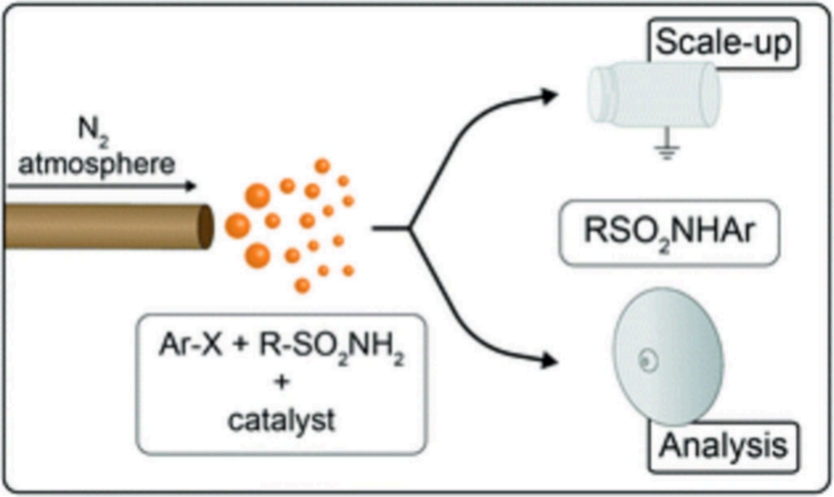
Microdroplet accelerated C–O and C–N coupling reactions

This was another collaboration where a student (K-Yi Iyer) spent time in our laboratories helping to develop our MS capabilities, particularly desorption electrospray ionization (DESI), which as mentioned earlier we also implemented for rapid HTE reaction analysis. The actual research studies were conducted in Graham Cooks’s laboratory. A future goal of this program would be to develop a large- scale micro droplet generator.

#### Summary

In this letter I have described several collaborative initiatives which met organizational goals in terms of developing fit for purpose enabling chemistry technologies including new synthetic methods using expertise and skill sets that were not available to us in house but resided in key academic groups. This was done primarily by either supporting students in our laboratories or supporting postdoctoral students in our partner’s academic laboratory.

Another important aspect of these efforts was the training of students in a “major Pharma” setting where they were exposed to an industrial working environment and high-level technologies. This helped prepare them for a subsequent career in industry. Notably, several students included part of their work in their PhD theses.

#### Safety

No unexpected or unusually high safety hazards were encountered.

## Abbreviations

C–O: carbon–oxygen bond
C–N: carbon–nitrogen bond
ES: Electrospray
PS: paper spray Mass Spectrometry
DESI: desorption electrospray ionization
GPCR: G-protein coupled receptor
HTE: high throughput experimentation
MCR: multiple component reaction
RCM: ring-closing metathesis
